# Pathogenesis and Molecular Immune Mechanism of Calcified Aortic Valve Disease

**DOI:** 10.3389/fcvm.2021.765419

**Published:** 2021-12-23

**Authors:** Weikang Bian, Zhicheng Wang, Chongxiu Sun, Dai-Min Zhang

**Affiliations:** ^1^Department of Cardiology, Nanjing First Hospital, Nanjing Medical University, Nanjing, China; ^2^Key Laboratory of Targeted Intervention of Cardiovascular Disease, Collaborative Innovation Center for Cardiovascular Disease Translational Medicine, Nanjing Medical University, Nanjing, China

**Keywords:** calcified aortic valve disease, aortic stenosis, inflammation, matrix remodeling, osteogenesis

## Abstract

Calcified aortic valve disease (CAVD) was previously regarded as a passive process associated with valve degeneration and calcium deposition. However, recent studies have shown that the occurrence of CAVD is an active process involving complex changes such as endothelial injury, chronic inflammation, matrix remodeling, and neovascularization. CAVD is the ectopic accumulation of calcium nodules on the surface of the aortic valve, which leads to aortic valve thickening, functional stenosis, and ultimately hemodynamic disorders. CAVD has become an important cause of death from cardiovascular disease. The discovery of therapeutic targets to delay or block the progression of CAVD and the clinical application of transcatheter aortic valve implantation (TAVI) provide new ideas for the prevention and treatment of CAVD. This article summarizes the pathogenesis of CAVD and provides insight into the future directions of CAVD diagnosis and treatment.

## Introduction

Valvular heart disease decreases activity tolerance of physical function and longevity. The death rate of rheumatic heart disease has remained fairly static since 2000, while deaths from calcific aortic stenosis continued to rise over the past 20 years (1). Calcified aortic valve disease (CAVD) is a common valvular disease that progresses from early valvular sclerosis without hemodynamic influence to severe calcified aortic valve stenosis requiring valve replacement (2). The main characteristics are inflammation, fibrosis, and calcification (3). In developed countries, calcified aortic valve disease is the most common cause of adult aortic valve stenosis, and the prevalence increases non-linearly with age. Currently, CAVD is considered to be an active and adjustable process accompanied by initiation factors that can promote disease occurrence, including clinical and genetic susceptibility and imbalances in molecules and cellular pathways (4).

Cardiovascular disease-related studies have shown that the incidence of all-cause death from cardiovascular disease caused by aortic valve sclerosis has increased by 35% (5). The early occurrence of aortic valve calcification affects patient prognosis and increases the risk of valve replacement surgery (6). As the most effective treatment for heart valve disease is valve replacement surgery, thus far, there has been no drug to treat or slow disease progression. At present, there is a lack of effective drugs for CAVD, so surgical replacement of calcified or stenotic valves is the only effective treatment (7). It is very important to determine the severity of the disease, analyze the calcification of the aortic valve, and assess the risk of cardiovascular disease to understand the opportunities and methods to improve patient prognosis (2). Based on these issues, this paper reviews the immune molecular process of CAVD and the effects of mechanical pressure and flow rate on the development of CAVD.

## Epidemiology and Clinical Characterization

According to the statistical committee of the American Heart Association (AHA) in the United States, the prevalence of CAVD in people over 65 years old is 20–30 and 48–57% in people over 85 years old; thus, CAVD has become the third most common cardiovascular disease after coronary heart disease and hypertension, and calcified aortic valve stenosis (CAS) has become the primary indication for aortic valve replacement (8). According to the results of a European Society of Cardiology (ESC) analysis, 81.9% of aortic stenosis in Europe is caused by calcification (9).

CAVD can be divided into primary and secondary CAVD. Primary CAVD is also known as senile aortic valve calcification or degenerative aortic valve calcification. Secondary CAVD calcification occurs on the basis of the original disease and is more common in rheumatic heart disease, infective endocarditis, dissecting aneurysm, congenital bicuspid valve, or single leaf aortic valve. Depending on the degree of aortic valve calcification, the condition can be divided into aortic valve calcification (AVC) and CAS. Under ultrasound evaluation, AVC shows valve thickening, strong echo shadows, valve orifice areas >3 cm^2^, cross valve blood flow rates <2.5 m/s, and unrestricted leaflet activity. With disease progression, in the context of AVC, the valve orifice area becomes narrower, the rate of cross valve blood flow becomes faster, and leaflet activity is limited; then, CAS occurs (10). Therefore, AVC is the early stage of CAS.

## Aortic Valve Calcification Process

**Figure 1:**
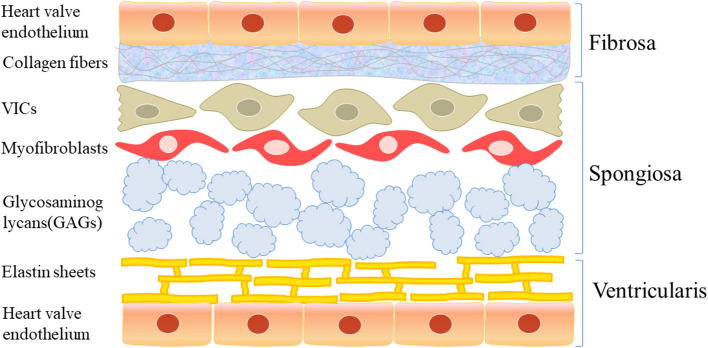
The structure of aortic valve. Macroscopically, the normal aortic valve is roughly divided into two edges, two sides, and three layers: free edge and basal edge, aortic surface and ventricular surface, ventricular layer, fibrous layer and sponge layer. Microscopically, it is mainly composed of valve endothelial cells and valve stromal cells and extracellular matrix such as collagen, glucan, and elastin.

Macroscopically, a normal aortic valve can be divided into two margins, two sides, and three layers: the free margin and basal margin, the aortic surface and ventricular surface, and the ventricular layer, fibrous layer, and spongy layer (Figure 1). Microscopically, the valve is mainly composed of valve endothelial cells, valve interstitial cells, and extracellular matrix components, such as collagen, dextran, and elastin.

Accumulating evidence has shown that calcified aortic valve disease is an active inflammatory disease caused by various factors. The pathological mechanism is complex and includes endothelial injury, inflammatory reactions, and oxidative stress that cause changes in cell composition in the valve, which is characterized by local thickening of the valve, the deposition of calcium salt, and the formation of calcium nodules, resulting in dysfunctional valve activity and hemodynamic changes.

The progression of calcified aortic valve disease is divided into two stages. The first stage is aortic valve sclerosis, in which extracellular matrix secretion is increased and some inflammatory cells infiltrate; the second stage is called aortic valve calcification, in which a large amount of calcium salts are deposited, forming calcium nodules, extracellular matrix components are abnormally increased, the valve leaflet is stiff and deformed, the number of interstitial cells in the valve is reduced, and neovascularization occurs (10–13).

## Pathogenesis

**Figure 2:**
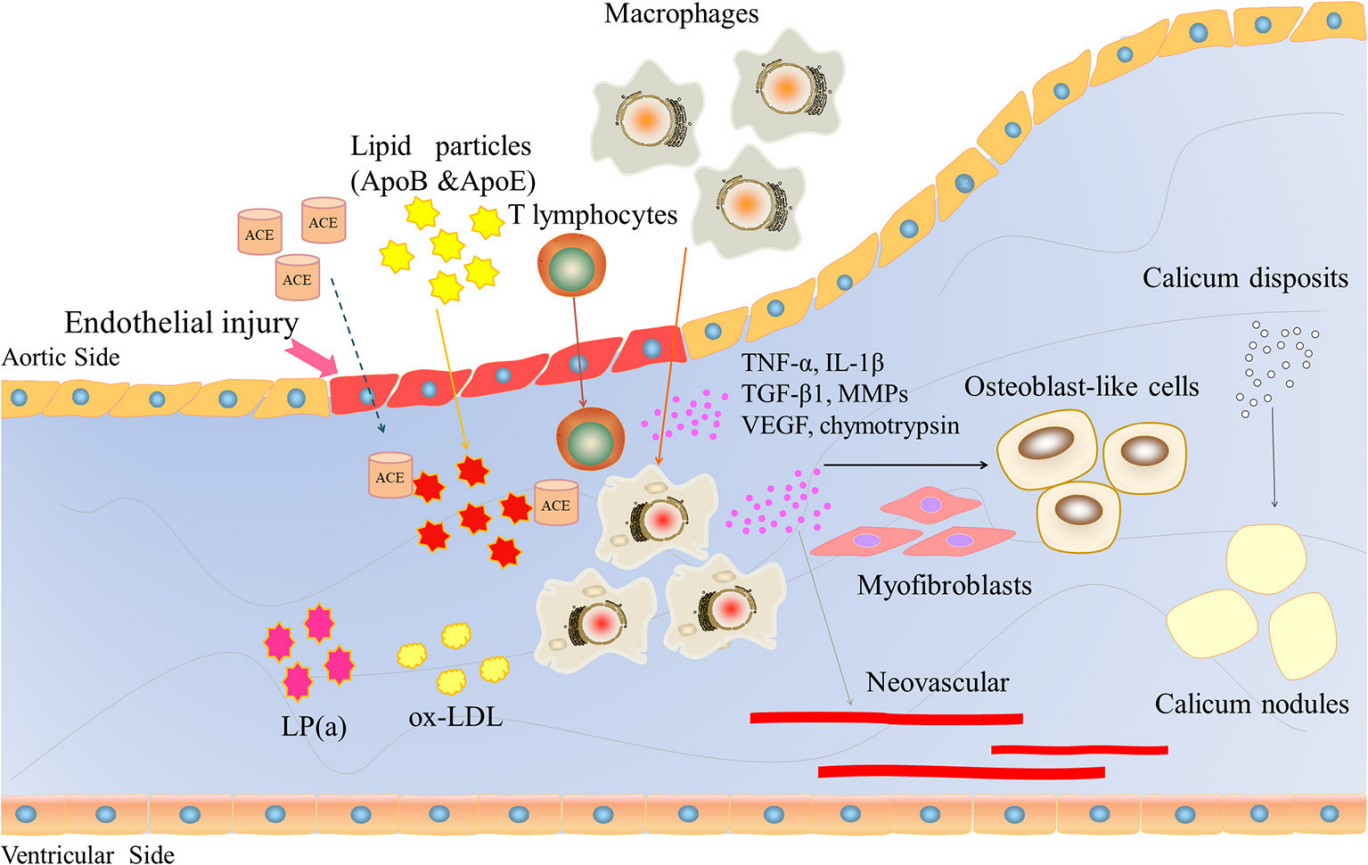
The role of inflammatory factors in regulating CAVD development and progression. Endothelial injury induces lipid particles (ApoB and ApoE), T lymphocytes and macrophages into the valve and releases large inflammatory factors, such as TNF-a, IL-1β, TGFβ1, and VEGF. VEGF induces neovascularization. These inflammatory factors lead to osteoblast-like cells, ultimately inducing calcium deposition.

CAVD is often associated with aging, degenerative changes, and calcium deposition and is regarded as an irreversible passive process. However, in recent years, it has been gradually recognized that CAVD is an active process involving endothelial injury, lipid infiltration, chronic inflammation, matrix remodeling, fibrosis, cell differentiation, progressive calcification, and neovascularization (Figure 2). Qiao et al. (14) identified severe hug genes and multiple potential miRNAs. These promising biomarkers and pathways for CAVD may provide novel molecular markers for diagnosis and targeted therapy. The pathological changes are similar to those of atherosclerosis in the early stage but may be similar to those of bone formation in the late stage.

### Endothelial Injury Caused by Mechanical Stress

The normal aortic valve is composed of three thin, smooth cusp valves. The specific physical and mechanical structure allows the valve to effectively adapt to the mechanical stress of blood flow. Therefore, under normal circumstances, most of the pressure on endothelial cells becomes laminar shear force to avoid endothelial cell damage. However, when the body exhibits abnormal conditions, such as a long-term increase in blood pressure or an increase in cardiac load caused by various reasons, a series of hemodynamic changes occur, such as turbulence, eddy currents, and other blood flow disorders. At this time, the stress on endothelial cells changes, and it is difficult for mechanical stress to become laminar shear force. Under the continuous impact of disturbed blood flow and the direct effect of mechanical stress, endothelial cells are damaged. The basement membrane is broken, damaging the protective barrier's function and resulting in dysfunction.

Structurally, the valves of patients with congenital bicuspid aortic valve malformation are composed of two cusp valves. Compared with the normal trilobal active valve, the bicuspid aortic valve will bear higher and unbalanced mechanical stress. Interestingly, in valve replacement and valve repair, most of the bicuspid aortic valves have calcifications. Studies have shown that on average, bicuspid aortic valve calcification occurs nearly 20 years earlier than tricuspid active valve calcification, and it very easily progresses to CAS (15). The most common site of aortic valve calcification is the fibrous layer of the aortic valve. The possible reason for this occurrence is that there is no blood flow in the mitral valve during diastole, while the fibrous layer of the aortic valve is adjacent to the aortic root, which is prone to hemodynamic changes such as turbulence. Common changes in the two valves are that the effect of laminar shear is reduced or even disappears, and the mechanical stress on endothelial cells increases (16). These findings provide the best evidence supporting the theory of endothelial injury.

### Lipid Infiltration Triggers Chronic Inflammation

Due to mechanical stress, after endothelial cell injury and basement membrane fracture, lipid components in the plasma will deposit at the fracture site and form scattered lipid points, and the main lipid components are apolipoproteins B and E. Renin-angiotensin converting enzyme (ACE) can be detected in the lesions, and ACE is usually associated with apolipoprotein B, which confirms that lipid particles bring ACE into the plasma (17). Endothelial cell injury and the formation of lipid points can trigger a chronic inflammatory response, attracting a large number of macrophages and a small number of T lymphocytes to gather near the lipid point through adhesion molecules. Immuno-histochemical staining and frozen section observation of diseased valves collected during surgery showed the presence of macrophages, which phagocytose mast cells formed by lipid transformation, scattered T lymphocytes, and a small number of smooth muscle cells (18, 19). A large amount of oxidized low-density lipoprotein (ox-LDL) and activated inflammatory cells could also be detected. The oxidized products are highly cytotoxic and stimulate inflammation and ossification in the later stage. Then, infiltrating inflammatory cells begin to act, releasing inflammatory factors, chemokines, growth factors, and cathepsin, as well as tumor necrosis factor α (TNF-α), transforming growth factor β1 (TGF-β1), vascular endothelial growth factor (VEGF), interleukin-1β (IL-1β), matrix metalloproteinase (MMPs), and chymotrypsin, which have been confirmed to be closely associated with calcification and stenosis of the aortic valve (20).

Cyclooxygenase (COX), also known as prostaglandin internal oxidase reductase, is a bifunctional enzyme with cyclooxygenase and catalase activities. It is the key enzyme that catalyzes the conversion of arachidonic acid to prostaglandins. Cyclooxygenase has two COX-1 and COX-2 isozymes. COX-1 mainly exists in blood vessels, the stomach, and kidney. It is involved in the regulation of vasomotor contraction, platelet aggregation, gastric mucosal blood flow, gastric mucus secretion, and renal function. COX1 is related to the protection of the gastrointestinal mucus membrane, the regulation of platelet aggregation, the regulation of peripheral vascular resistance and the regulation of renal blood flow distribution. COX2 is inducible. Various damaging chemical, physical and biological factors activate phospholipase A2 to hydrolyze cell membrane phospholipids to produce arachidonic acid. The latter generates prostaglandins through COX-2 catalytic oxygenation. In normal tissue/cells, the activity of COX-2 is very low. When cells are stimulated by inflammation, their expression level in inflammatory cells can increase to 10–80 times the normal level, resulting in an increase in the contents of PEG2, PGI2, and PGE1 in inflammatory sites and resulting in an inflammatory response and tissue damage. Vieceli Dalla Sega et al. (21) reported that COX-2 expression in human aortic valve interstitial cells (AVICs) is decreased in patients with calcific valves. Celecoxib, a COX-2 inhibitor, induced AVICs toward a myofibroblast phenotype and increased the expression of TGF-β to promote the formation of calcific nodules. These data support that celecoxib may facilitate CAVD progression. Treatment with celecoxib increased dystrophic calcification of aortic valve interstitial cells *in vitro*, while dimethyl celecoxib prevented TGF-β1-mediated calcification of AVICs (22).

### Matrix Remodeling and Fibrosis

Under the stimulation of inflammatory mediators, chemokines, growth factors, and cathepsins released by inflammatory cells, normal collagen fibers and elastic fibers in the aortic valve are destroyed. Furthermore, fibroblasts in the aortic valve are induced to differentiate into myofibroblasts and are activated for a long time, producing a large amount of collagen. The interstitial layer of the aortic valve was originally composed of type I and type III collagen. The regular arrangement of fibers and elastic fibers is destroyed. Furthermore, the ratio of collagen and elastin is disrupted, extracellular collagen levels are increased, and the matrix is reconstructed, which results in continuous thickening and weakened elasticity in the valve. Finally, the thin, elastic valve becomes fibrous, thickened, and hardened, and loses elasticity (17). MMPs and tissue inhibitors of matrix metalloproteinases (TIMPs) play important roles in this process. In normal tissues, there is a dynamic balance between MMPs and TIMPs to maintain extracellular matrix homeostasis. In the calcified valve interstitium, this balance is completely broken. The expression of MMP-1, MMP-2, and MMP-9 was significantly higher than that in the normal valve. *In vitro* cell experiments showed that the activity of MMP-2 was regulated by inflammatory factors (TNF-α, IL-1β) (20), which further verified that matrix remodeling was a pathological change in response to chronic inflammation.

### Cell Differentiation, Calcification, and Bone Formation

Myofibroblasts in the aortic valve are phenotypically between smooth muscle cells and fibroblasts and have dual properties. Under the stimulation of TGF-β1, bone matrix protein and other cytokines, myofibroblasts differentiate into osteoblast-like cells, and then heterotopic calcification occurs in the valve. Active osteogenic changes and bone resorption can be observed. With disease development, even messy lamellar bone tissue can appear. Recently, a large-scale clinical study analyzed valves removed from CAS patients and found that more than 10% of patients had bone or cartilage formation in valves, and the ossification in cartilage was similar to the repair of normal fractures (21). In addition, the theory of myofibroblast differentiation into osteoblast-like cells has been confirmed at the cell culture and molecular levels. During the process of bone formation, a variety of signaling molecules and signal transduction pathways are involved. Bone matrix proteins, including osteopontin (OPN), bone sialoprotein (BSP), bone morphogenetic protein (BMP), osteocalcin (OCN), and alkaline phosphatase (ALP), are markers of osteoblast differentiation and maturation and are closely related to bone formation. These factors are significantly increased in the valve tissues of patients with CAVD (22), while some miRNAs that can inhibit the expression of OPN and BMP, such as miRNA-141, are significantly reduced in the diseased valve (23).

### Angiogenesis

There are no microvessels in normal heart valves. The nutrients and oxygen needed by valve cells are mainly supplied by the diffusion of blood. However, when the valve exhibits inflammatory infiltration and ossification, the delicate balance between angiogenic factors and vascular inhibitors is broken, resulting in the formation of new microvessels. Studies have shown that when valve lesions occur, the expression of VEGF and its receptor, secreted protein acidic and rich in cysteine (SPARC) and bone connexin, is greatly increased, and the expression of the vascular growth inhibitor chondromodulin-1 is decreased, which eventually leads to the formation of new blood vessels (24–26). Neovascularization not only creates conditions for the infiltration of inflammatory cells and plasma lipids but also promotes endochondral osteogenesis in valves (17). However, we all know that there are no blood vessels in normal valves, so it is worth discussing where the endothelial cells in new microvessels come from. Human valve interstitial cells (VICs) are involved in aortic valve angiogenesis. These effects were inhibited by blocking VEGF-A with a blocking antibody or siRNA in a VEGF-A-dependent mechanism (27).

## The Influence of Mechanical Pressure and Flow Rate on the Development of CAVD

The aortic valve is mechanically stimulated by shear stress, strain, and tension/pressure in lobular tissue. The aortic valve mainly faces two types of pressure: oscillatory flow from the fibrous membrane surface of the valve and laminar flow from the ventricular muscle surface (28). The fibrous membrane of the valve is mainly subjected to oscillatory shear and is more prone to calcification than the ventricular muscle, which is sensitive to laminar shear. Simmons et al. (28) showed that endothelial cells passing through the fibrous membrane could express osteogenic factors, while cells in ventricular muscle could express calcification inhibitors. However, it is not clear whether the diversity of the valve endothelial cell (VEC) population and location and origin of VEC development affect mRNA expression under different biomechanical pressures. Using oscillatory flow and laminar flow models, Moore et al. (29) verified the specificity of aortic anatomy in different patients, and these differences could produce different flow patterns, which were related to the formation of local calcium nodules. These findings may explain the dramatic changes in flow rate in some populations, including patients with bicuspid aortic valve (BAV) who have a bicuspid rather than a normal tricuspid valve. Based on a computational growth and remodeling (G&R) framework, Sadrabadi et al. (30) developed an algorithm to evaluate the effects of aging and calcification on aortic valve dynamics. The patterns in geometric orifice area reduction and an increase valve stress during local and global growth and remodeling of the aortic valve.

In the pathological progress of calcific aortic valves, glycosaminoglycans (GAGs) shift from the valve spongiosa to the collagen I-rich fibrosa layer near calcified nodules. In a complete model of aortic valve disease, including endothelial cells, interstitial cells, and disease-like ECM, the glycosaminoglycan chondroitin sulfate (CS) increases the expression of pro-calcific genes and calcified nodule formation (31).

Fisher et al. (32) used the Flexcell tension system to place VICS in a tension environment, used TGF-β1 to treat calcified nodules, and compared the outcomes to the effect of no pressure (with TGF-β1 treatment). The finite element method also showed that mechanical tension has a positive regulatory effect on calcification (33). However, it is not clear whether mechanical pressure can initiate changes in valve shape and stiffness before osteogenesis or whether other small changes lead to changes in the flow pattern.

## The Role of Clonal Hematopoiesis of Indeterminate Potential (CHIP)

Clonal hematopoiesis of indeterminate potential (CHIP) is a blood disorder with some gene variants from a hematopoietic stem cell. CHIP itself does not denote a malignancy, but the individual with CHIP associates with higher risks of malignant blood disorders and atherosclerotic cardiovascular diseases. The threshold for CHIP was set a variant allele fraction of 2%. *In vitro* evaluation of Tet2, a gene known to have a role in inflammation regulation, germline sites may help to identify mutations that interfere with the function of the Tet2 distal enhancer. Disrupting the Tet2 distal enhancer will increase the self-renewal level of hematopoietic stem cells. Mutations in germline genetics shape hematopoietic stem cell function and induce CHIP formation (34).

Mas-Peiro et al. (35) reported the incidence of CHIP increased in an age-dependent manner. Patients with DNMT3A or Tet2-CHIP driver mutations had significantly increased medium-term all-cause mortality following TAVI. Mutation of CHIP driver genes is associated with increased pro-inflammatory leucocyte subsets and increased mortality following successful TAVI. Recent evidence has shown that CHIP has an increased risk of developing cardiovascular disease (36). A study observed eight patients with severe degenerative aortic value stenosis, six with chronic post-infarction heart failure, and three normal participants (37). Patients who carry CHIP driver sequence variations and have cardiovascular disease are susceptible to a greater inflammatory response.

The role of inflammation in cardiovascular events has always been a research hotspot. Recently, a large prospective randomized clinical trial (Cantos) conducted anti-inflammatory therapy targeting interleukin-1β in patients with existing myocardial infarction and continuous increase of inflammatory marker C-reactive protein. The comparative study of monoclonal antibody canakinumab and placebo showed that patients in the intervention group had fewer recurrent events (38). The enrolled samples of patients in the study are being tested to determine whether those with CHIP benefit the most from the intervention. If so, it means finding a potential tool to reduce the vascular risk of CHIP. Considering the prevalence of CHIP in the elderly and the high incidence of stroke and myocardial infarction, CHIP vascular risk is a greater public health risk than hematological malignancies.

## CAVD Treatment

In CAVD, the main treatment strategy is to control the related pathogenic factors, such as antihypertension, smoking cessation, and body weight control. Although a small retrospective analysis showed that the use of bisphosphonates for anti-osteoporosis treatment and ACEI drugs to inhibit the effect of angiotensin could delay the progression of CAVD (39, 40), there is a lack of large-scale prospective clinical trials, and the mechanism and target effect of bisphosphonates need to be strengthened. Surgical valve replacement remains the main treatment for CAVD patients with obvious symptoms or very severe asymptomatic CAS patients. Recent evidence has shown that transcatheter aortic valve replacement (TAVR) is expected to become a treatment for CAVD patients who cannot tolerate surgery (41, 42). TAVR is rapidly expanding the treatment of CAVD and bicuspid aortic valves (43). Evogliptin inhibits CAVD by reducing inflammation, fibrosis, and calcification, suggesting its potential as a targeted therapeutic agent for inhibiting CAVD progression (44).

Although aortic valve replacement has been shown to be effective in the late stage of CAVD, it is costly and not optimal for elderly patients. These issues drive increasing interest in non-invasive therapies for patients with CAVD. Macrophages, T lymphocytes, and B lymphocytes are increased in the aortic valve with the progression of valve calcification, as well as cytokine signaling. This evidence indicates that modulation of adaptive immune cell signaling may be a strategy for treating CAVD (45). With further understanding of the molecular immune mechanism of CAVD, well-designed drugs for treating CAVD will bring more hope to patients.

## Conclusion and Prospects

An increasing number of studies have begun to find new immune molecules associated with the pathogenesis of CAVD and explore the role of mechanical pressure and flow on disease occurrence. Although elucidating the pathogenesis of CAVD is helpful for treatment, its biological complexity remains the main challenge for individualized therapy.

The pathophysiological changes in CAVD are similar to those in atherosclerosis in the early stage but may be similar to those in bone formation in the late stage and involve abnormal signal transduction pathways and gene variation. However, the promoting factors, the relationship between each pathway, and the specific molecular mechanism need to be further studied. CAVD pathogenesis can be summarized as endothelial injury, lipid infiltration, and chronic inflammation. Of course, the development and continuous improvements in TAVI technology, as well as the discovery of new and effective therapeutic targets, have brought hope to patients. However, more clinical trials and long-term follow-up are needed to further verify this hypothesis. It is believed that with the consistent efforts of researchers, breakthroughs will be made in both basic research and control methods of CAVD in the future.

## Author Contributions

WB and ZW contributed to literature search. DMZ and CS drafted the manuscript and contributed to review design. All authors contributed to the article and approved the submitted version.

## Funding

This work was supported by the National Natural Science Foundation of China (81870355 and 81970342) and Jiangsu Provincial Key Research and Development Program (BE2018611).

## Conflict of Interest

The authors declare that the research was conducted in the absence of any commercial or financial relationships that could be construed as a potential conflict of interest.

## Publisher's Note

All claims expressed in this article are solely those of the authors and do not necessarily represent those of their affiliated organizations, or those of the publisher, the editors and the reviewers. Any product that may be evaluated in this article, or claim that may be made by its manufacturer, is not guaranteed or endorsed by the publisher.
